# A New Inductive Debris Sensor Based on Dual-Excitation Coils and Dual-Sensing Coils for Online Debris Monitoring

**DOI:** 10.3390/s21227556

**Published:** 2021-11-13

**Authors:** Xianwei Wu, Yinghong Zhang, Nian Li, Zhenghua Qian, Dianzi Liu, Zhi Qian, Chenchen Zhang

**Affiliations:** 1 State Key Laboratory of Mechanics and Control of Mechanical Structures, College of Aerospace Engineering, Nanjing University of Aeronautics and Astronautics, Nanjing 210016, China; 18335166122@163.com (X.W.); yh_zhang@guet.edu.cn (Y.Z.); nianli@nuaa.edu.cn (N.L.); qianzhi2021@nuaa.edu.cn (Z.Q.); zhangcc@nuaa.edu.cn (C.Z.); 2 School of Engineering, University of East Anglia, Norwich NR4 7TJ, UK; dianzi.liu@uea.ac.uk

**Keywords:** inductive debris sensor, online debris monitoring, metal debris

## Abstract

Lubricants are of key importance for mechanical processing, and exist in nearly every mechanical system. When the equipment is in operation, debris particles will be generated in mechanical lubricants. The detection of debris particles can indicate the wear degree of machinery components, and provide prognosis warning for the system before the fault occurs. In this work, a novel type of inductive debris sensor consisting of two excitation coils and two sensing coils is proposed for online debris monitoring. The developed sensor was proven to be of high sensitivity through experimental verification. The testing results show that, using the designed sensor, ferrous metal debris with a size of 115 μm and nonferrous metal debris with a size of 313 μm in a pipe with an inner diameter of 12.7 mm can be effectively detected. Moreover, the proposed inductive debris sensor structure has better sensitivity at higher throughput and its design provides a useful insight into the development of high-quality sensors with superior performances.

## 1. Introduction

Fault detection and condition monitoring of machines are important methods to maintain operational performance and extend the service life of rotating and reciprocating machinery in many sectors such as machinery manufacturing, the transportation industry, and the military. The applications of these technologies can prevent the breakdown of critical system components and avoid unexpected production delays [1]. Detecting metal debris in the lubrication oil is a direct and dependable method for monitoring the condition of rotating and reciprocating machinery [2,3,4]. Under normal operating conditions, the metal debris retains a stable size and concentration in the lubrication oil. However, when there is abnormality then the concentration and size of metal debris will increase [5,6,7]. Taking into account this situation, the real-time monitoring of the working condition of mechanical equipment has attracted increasing attention from researchers. Since the real-time online detection of metal debris in the lubricating oil is an important task, several new techniques and methods have been developed in recent decades to improve the accuracy of debris detection.

In general, the existing detection techniques, including various online and off-line inspection methods, can be divided into the following six classes: optical scattering counter method [8,9], capacitance method [10], resistance method [11], ultrasonic method [12], X-ray method [13], and inductive method [14,15,16]. Different detection methods have different advantages, and undoubtedly have some limitations which constrain their industrial utilizations. For example, the reliability of the optical method is quite poor because it requires the transparency of both the oil and inclusive bubbles. The application of capacitance or resistance methods will induce oil deterioration, which will degrade the detection accuracy as time goes. The accuracy of the ultrasonic method is affected by the viscosity of the oil, the flow rate, and mechanical vibration, which is hard to eliminate in practical applications. The X-ray method has high detection precision, but demands complex equipment. The inductive method is suitable for both metal and non-metal pipelines and the associated equipment has a simple structure. Moreover, the sensitivity of this method does not rely on the oil quality, and it can effectively distinguish non-ferrous and ferrous metal debris. However, it has some limitations, including the low sensitivity to non-ferrous metal debris and the incapability of detecting debris shape. From a practical point of view, the inductive method is the most feasible and effective technique for many applications.

Since the inductive method has many advantages, a lot of studies have been conducted by researchers in this field. Flanagan et al. [17] first proposed a method for testing debris material and size with a single-coil sensor in 1990. Experimental results showed that the sensor can effectively detect debris of 100 μm in a pipe with a 6-mm diameter. In industrial applications, MetalSCAN from GasTop is a widely used sensor. It consists of one induction coil and two excitation coils around the same tube. The specifications of the MetalSCAN product indicate that its sensitivity to ferrous and non-ferrous metal debris in the inner diameter of the pipe, which was approximate 9.525 mm [18], could be achieved with values of 100 μm and 405 μm, respectively. One problem that remains to be solved is that the detection performance of this sensor is seriously affected by background noise and vibration signals. Talebi et al. [19] designed the sensor to effectively detect 125 μm ferrous debris in pipes with an internal diameter of 4 mm, and it can detect the concentration of metal debris in the oil. However, the 4 mm-diameter of the pipe limits the flow rate of the oil. In order to improve the accuracy of detection, Ren et al. [20] proposed a sensor using an excitation coil and two induction coils. It can identify the 120 μm ferrous debris and 210 μm non-ferrous debris in a 34 mm-diameter pipe. However, the induction coil should be immersed into the oil, which will result in increased resistance in the flow of lubricants. Du et al. [21,22,23] made improvements to the original basis of the sensor using the parallel LC resonance method. The sensor’s sensitivity was obviously improved with the ability to detect the 20 μm debris. Its excellent performance benefited from the use of a microfluidic channel with a diameter of 250 μm. The practical application of this sensor is still limited because the micro-size of the channel leads to the blockage. Also, a considerable throttling effect, which results in the unsuitability of the sensor to high-rate flow tests, exists in the channel.

In order to develop a high-sensitive sensor that is suitable for the high-rate flow test, a novel sensor design consisting of two excitation coils and two sensing coils has been proposed in this paper. To prove the sensitivity and applicability of the developed sensor, experimental tests have been conducted to demonstrate its superior performance.

## 2. Sensor Principle Design

**Figure 1:**
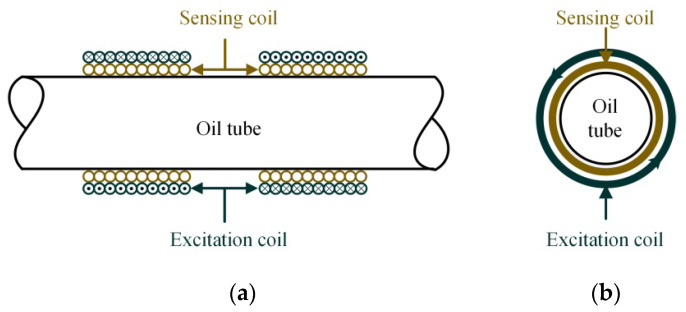
The structure of the new designed inductive debris sensor: (**a**) the front view of the sensor; (**b**) the side view of the sensor.

The mechanical structure of the sensor is mainly composed of two excitation coils and two sensing coils. The two sensing coils are placed side by side, with two sides being symmetrical, and the two excitation coils are arranged right outside the two sensing coils, as shown in Figure 1.

**Figure 2:**
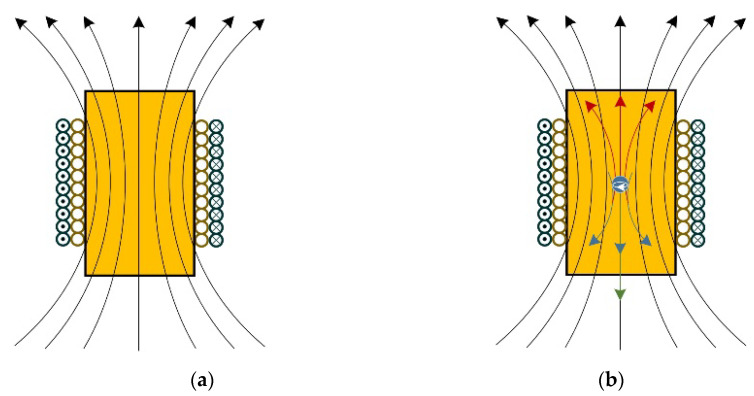
The magnetic field distribution of the sensor-designed sensor: (**a**) no metal debris flows through; (**b**) when ferrous metal debris enters the sensor.

The sensor’s operating principle is as shown in Figure 2. An AC voltage is applied to the excitation coils, which generates the magnetic field as shown in Figure 2a. When ferrous metal debris enters the sensor, two factors (permeability and eddy current) will interact with each other, as shown in Figure 2b. First, the magnetic flux will increase due to the higher permeability of the ferrous metal debris. Second, a magnetic field whose direction is opposite to the original magnetic field will be generated by the eddy currents inside the ferrous metal debris, which will decrease the total magnetic flux. At low frequency, the increase of magnetic flux dominates, which means a positive voltage pulse will be generated when ferrous metal debris flows through the sensor.

## 3. Mathematical Modeling of Sensors

According to Biot-Savart’s theorem, the magnetic field of a circular current-carrying wire is [24,25].

(1) $$B=\frac{\mu_{0}Ir^{2}}{2{\left(r^{2}+x^{2}\right)}^{\frac{3}{2}}}$$

where B is the magnetic field strength of the circular current-carrying wire at the target point, $\mu_{0}$ is the vacuum magnetic permeability, I is the excitation current, r is the radius of the circle, and x is the transverse coordinate of the target point.

**Figure 3:**
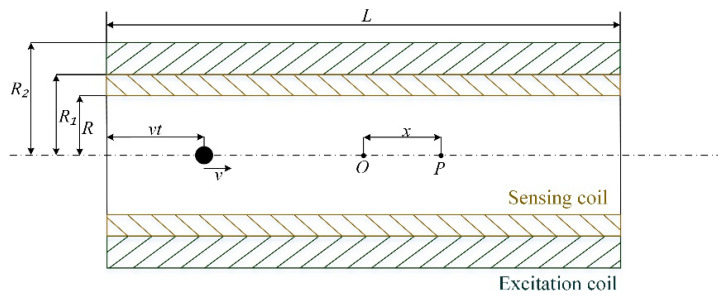
The diagram of the sensor.

The sensor’s parameter model is shown in Figure 3. Where $n_{1}$ is the number of turns per unit length of the excitation coil, $R_{1}$ is the inner diameter of the excitation coil, $R_{2}$ is the outer diameter of the excitation coil, $N_{1}$ is the number of turns of the excitation coil, R is the inner diameter of the sensing coil, $N_{2}$ is the number of turns of the sensing coil, I is the amplitude of the excitation signal, L is the length of the sensing coil, the midpoint of the excitation coil is set as the origin, x is the axial distance.

Generally, inductive sensors are composed of multiple layers of solenoids. The central axis of the solenoid is set as the origin. The magnetic field at any point on the axis of the multi-layer solenoid is represented as follows.

(2) $$B=\frac{\mu_{0}n_{1}I}{2}\left[-\left(x-\frac{L}{2}\right)\ln\frac{R_{2}+\sqrt{R_{2}^{2}+{\left(x-L/2\right)}^{2}}}{R_{1}+\sqrt{R_{1}^{2}+{\left(x-L/2\right)}^{2}}}+\left(x+\frac{L}{2}\right)\ln\frac{R_{2}+\sqrt{R_{2}^{2}+{\left(x+L/2\right)}^{2}}}{R_{1}+\sqrt{R_{1}^{2}+{\left(x+L/2\right)}^{2}}}\right]$$

Assuming that the metal debris particles are spherical with radius $r_{a}$, the change in axial magnetic flux when metal debris enters the sensor is [23]

(3) $$d\varphi =dB\cdot S=\left(\mu_{r}-1\right)\pi R^{2}BV_{0}$$

where $V_{0}$ is the volume of the metal debris, $V_{0}=4/3\pi r_{a}^{3}$, according to the principle of electromagnetic induction, can be obtained from the sensing coil generated by the induction electromotive force:

(4) $$E=-N_{2}\left(\mu_{r}-1\right)\pi R^{2}V_{0}\frac{dB}{dt}$$

The excitation signal is a sinusoidal AC current i=Icos(2πft), and the induced electric potential is

(5) $$E=-N_{2}\left(\mu_{r}-1\right)\pi R_{1}^{2}V_{0}\frac{\mu_{0}n_{1}}{2}\left(i\frac{dK}{dt}+K\frac{di}{dt}\right)$$

where

(6) $$K=\left[-\left(x-\frac{L}{2}\right)\ln\frac{R_{2}+\sqrt{R_{2}^{2}+{\left(x-L/2\right)}^{2}}}{R_{1}+\sqrt{R_{1}^{2}+{\left(x-L/2\right)}^{2}}}+\left(x+\frac{L}{2}\right)\ln\frac{R_{2}+\sqrt{R_{2}^{2}+{\left(x+L/2\right)}^{2}}}{R_{1}+\sqrt{R_{1}^{2}+{\left(x+L/2\right)}^{2}}}\right]$$

Assuming that the velocity of the metal debris through the sensor is v, the position of the metal debris is x=vt−L/2, and the induced electric potential is

(7) $$E_{1}=-\frac{2N_{1}N_{2}\left(\mu_{r}-1\right)\mu_{0}\pi^{2}R_{1}^{2}r_{a}^{3}I}{3L\left(R_{2}-R_{1}\right)}\left[\cos\left(2\pi ft\right)\frac{dK}{dt}-2\pi fK\sin\left(2\pi ft\right)\right]$$

Since the two sets of coils have the same structure, when the metal debris passes through the second set of coils, the induced electric potential is

(8) $$E_{2}=-\frac{2N_{1}N_{2}\left(\mu_{r}-1\right)\mu_{0}\pi^{2}R_{1}^{2}r_{a}^{3}I}{3L\left(R_{2}-R_{1}\right)}\left[\cos\left(2\pi f\left(t-\Delta t\right)\right)\frac{dK}{dt}-2\pi fK\sin\left(2\pi f\left(t-\Delta t\right)\right)\right]$$

The time difference between the metal debris passing through the two sets of coils is Δt=(L+d)/v, where d is the distance between the two sets of coils. The induced electric potential output from the sensor is

(9) $$E=E_{1}-E_{2}$$

**Figure 4:**
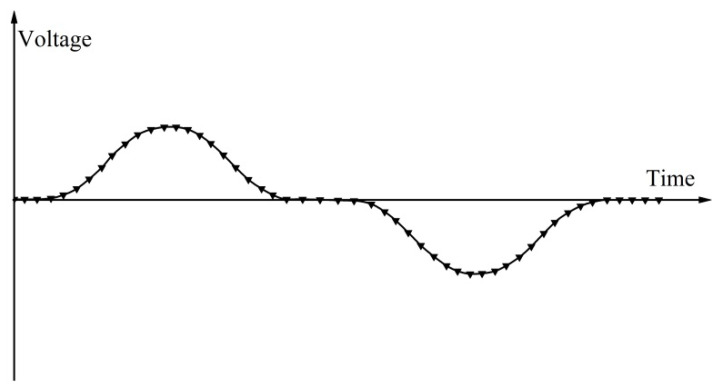
The curve of induction electromotive force of the mathematical model.

We can obtain the curve of the induced electrostatic force according to Equation (9) as shown in Figure 4.

## 4. Experimental Process

### 4.1. Design of the Sensor

The manufacturing process of the designed four-coil structure is briefly introduced below. First, make the sensing coil and wind 0.1 mm diameter enameled wire on an epoxy resin skeleton (Wuxi Petrochemical Equipment Co, Wuxi, China) with an inner diameter of 12.7 mm and a thickness of 1 mm (because the magnetic permeability of epoxy resin is close to that of air, the epoxy resin has a small effect on the magnetic field), with a total of 4 layers of winding and 200 turns per layer. Then, make the excitation coil by winding 0.2 mm diameter enameled wire around the outside of the sensing coil, with a total of 4 layers and 100 turns per layer.

### 4.2. Signal Processing Method

**Figure 5:**
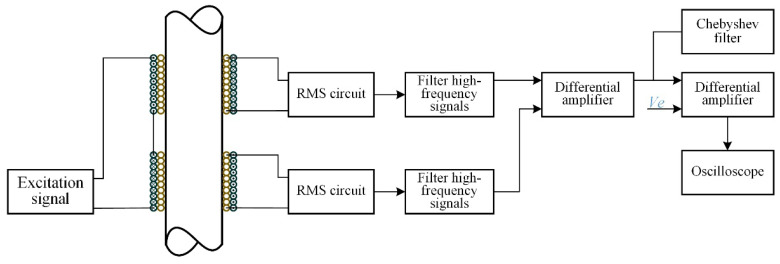
Sensor signal acquisition and processing system.

In order to extract the accurate response signal of metal debris, and reduce the high-frequency noise disturbance to a minimum degree, the output voltage of the sensor sensing coil, a simple and effective signal acquisition, and a processing system are designed in our work, as shown in Figure 5. A sinusoidal signal of ±10 V and 125 kHz is generated as the excitation signal of the sensor system (Through experiments, we know that the sensor has the highest sensitivity when the excitation frequency is 120–130 kHz, this will be confirmed later). In the sensing coil, a sinusoidal signal with the same frequency as the excitation signal is then induced. When metal debris passes through the sensing area, a signal will be generated correspondingly, which, however, is very weak and emerges with the induced sinusoidal signal. The variation of the signal arising from the metal debris is hard to detect directly and so a signal processing system is needed.

Firstly, the AC signal is converted to a DC signal by true RMS conversion (“true RMS conversion” means the process in which the full-wave rectification of a sinusoidal signal is followed by low-pass filtering, then the signal is converted to a DC signal). The DC signal is then differentially amplified using the low-noise amplifier INA114 with a gain of G = 500 (The INA114 is made by Texas Instruments, Inc., Dallas, TX, USA). Due to errors arising from coil processing, the two DC signals are slightly different. After differential amplification, there presents a nonzero signal called bias voltage, which will affect the next step of the amplification effect. A compensation voltage (*Ve*) is introduced during the second amplification to balance the bias voltage. Still, INA114 low-noise amplifier is adopted, with gain G = 100. It uses a Chebyshev filter to improve the signal-to-noise ratio. The oscilloscope shows the final output results. A signal similar to a cycle of a sine function will be detected when metal debris passes through the sensing area of the sensor.

### 4.3. Experimental Setup

**Figure 6:**
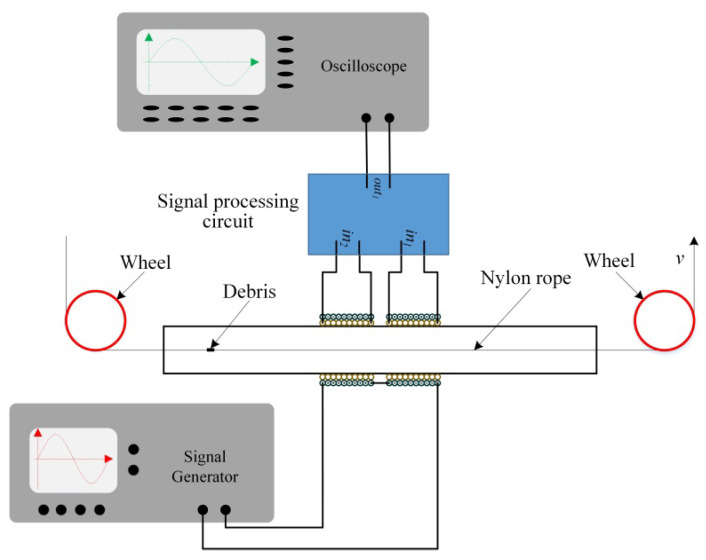
Schematic diagram of the experimental platform.

The schematic diagram of the experimental platform is shown in Figure 6. To be able to accurately control the speed and position of the metal debris passing through the sensor area, the metal debris can be fixed in the nylon rope. Additionally, the nylon rope is driven by a motor, and the moving speed of the nylon rope is controlled by controlling the speed of the motor, then controlling the speed and position of the metal debris through the sensor area. The nylon’s permeability is close to that of air, so the nylon rope has a small effect on the magnetic field. In practice, the shapes of metal debris produced by the mechanical wear process are not consistent, which causes difficulty for experimental analysis. In order to better quantify the experimental results, in our work, nearly spherical metal debris is used in the experiment.

## 5. Experimental Results and Discussion

### 5.1. Experimental Result

**Figure 7:**
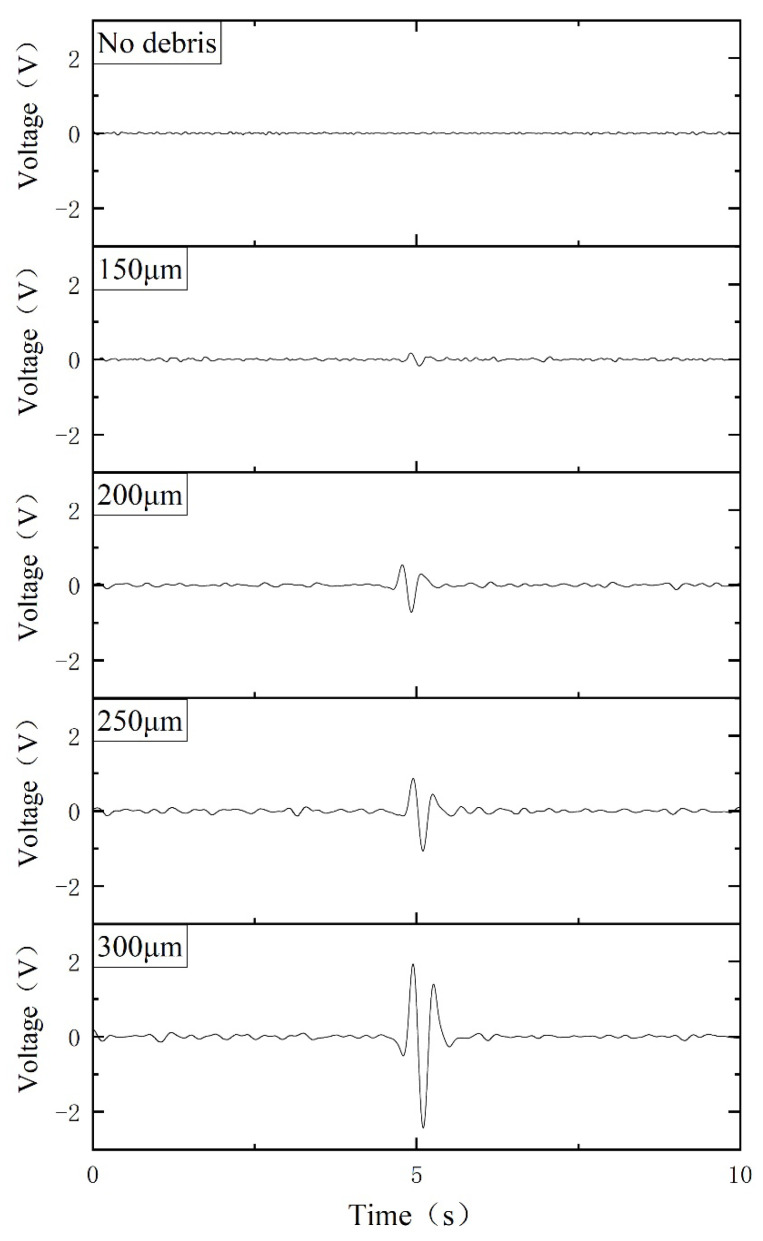
Voltage signals are generated by the passage of ferrous metal debris of different diameters.

**Figure 8:**
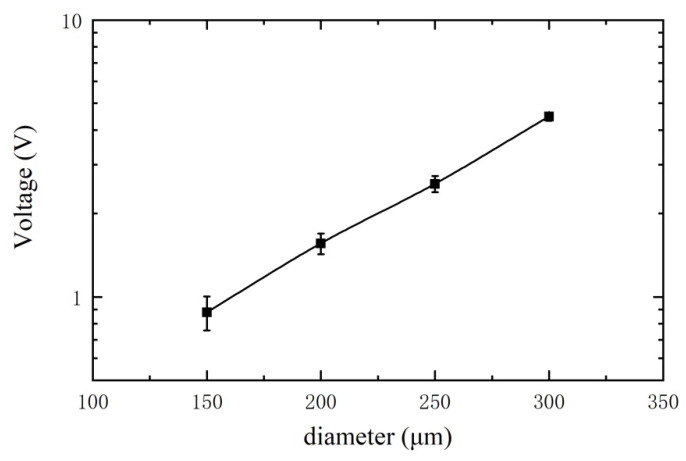
Variation of output voltage with metal debris size.

For experimental comparison study, a series of ferrous metal debris is selected, with diameters of 150 μm, 200 μm, 250 μm, and 300 μm respectively (the tolerance is approximately ±10 μm). The excitation signal is ±10 V and 125 kHz. The velocity of the metal debris passing through the sensor is fixed as 0.2 m/s. The final output signals of the corresponding metal debris are shown in Figure 7. The first graph shows the noise level of the sensor without metal debris passing through. An obvious output signal (greater than the background noise voltage) can be observed when ferrous metal debris with a diameter of 150 μm passes through the sensor, which means the designed sensor can effectively detect the ferrous metal debris with a diameter larger than 150 μm. The amplitude of output voltage correspondingly increases with the increase in the diameter of metal debris. The relationship between metal debris size and the output voltage is shown in Figure 8 (where the size of each metal debris particle is counted using 12 sets of experimental data, and the short line indicates the standard deviation), and the output voltage is proportional to the volume of the metal debris, as can be derived from Equation (7). Based on this law, we can determine the size of the metal debris by detecting the output voltage value. Since the output voltage signal is proportional to the debris volume, it can be deduced that the detection limit of the sensor is 150 μm ÷ $\sqrt[{3}]{\frac{880}{400}}$ ≈ 115 μm (The magnitude of the noise included in the circuit is 400 mV, and the magnitude of the output voltage is 880 mV when a ferrous metal debris particle with a diameter of 150 μm passes through the sensor).

### 5.2. Sensor’s Frequency Characteristic

**Figure 9:**
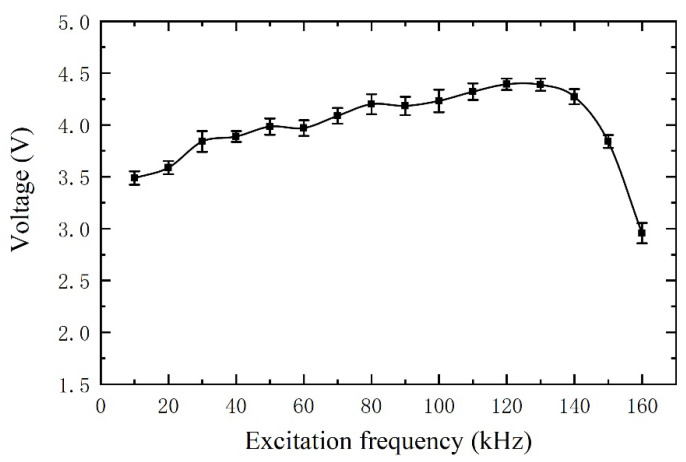
The sensor’s frequency characteristic.

For inductive sensors, the excitation frequency is also one of the key factors affecting the sensitivity of the sensor. A group of experiments is carried out to study the influence of excitation frequency on the sensor’s sensitivity, which selected 300 μm ferrous metal debris for the experiment. The speed of metal debris passing through the sensor is still fixed as 0.2 m/s, and the excitation signal voltage is ±10 V. The experimental results are shown in Figure 9 (all the experiments are repeated 12 times, and the values shown in the figure take an average of the 12 tested values, and the short line indicates the standard deviation). The experimental results show the sensor’s sensitivity reaches the maximum when the excitation frequency is 120–130 kHz.

### 5.3. Influence of Radial Distribution of the Magnetic Field on Sensitivity

**Figure 10:**
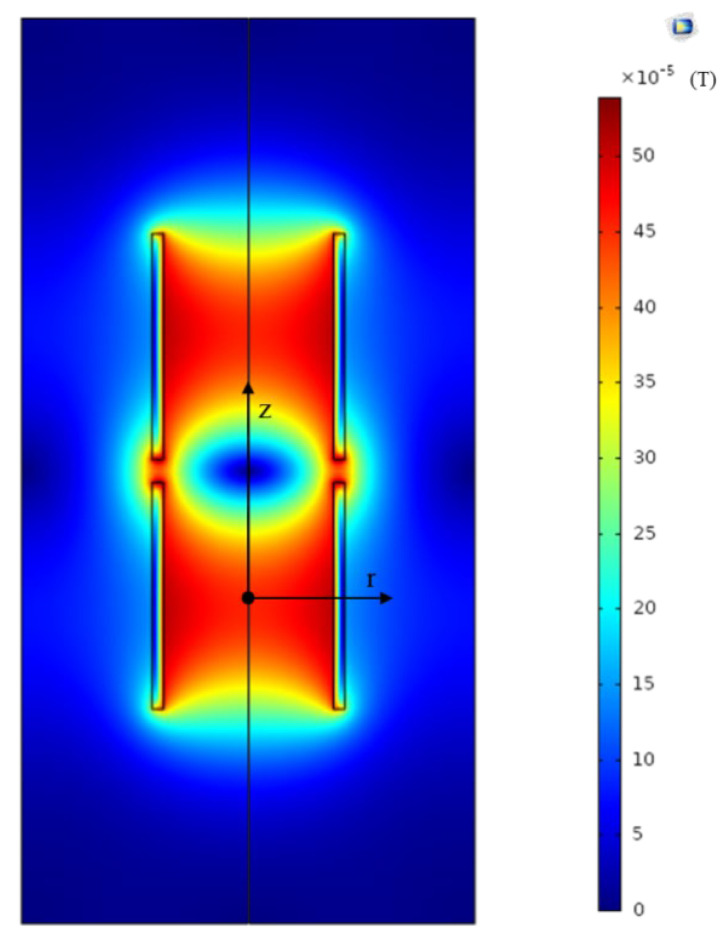
The magnetic flux density distribution of an excitation coil.

**Figure 11:**
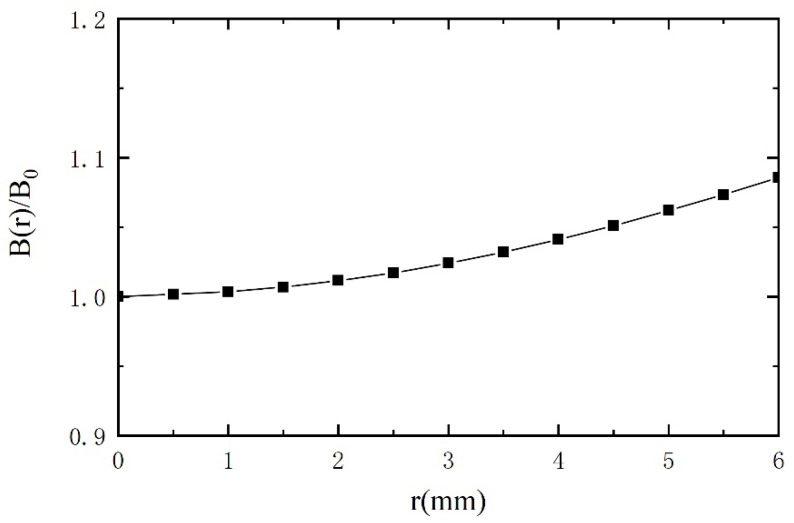
Radial distribution of relative magnetic flux density at z = 0.

**Figure 12:**
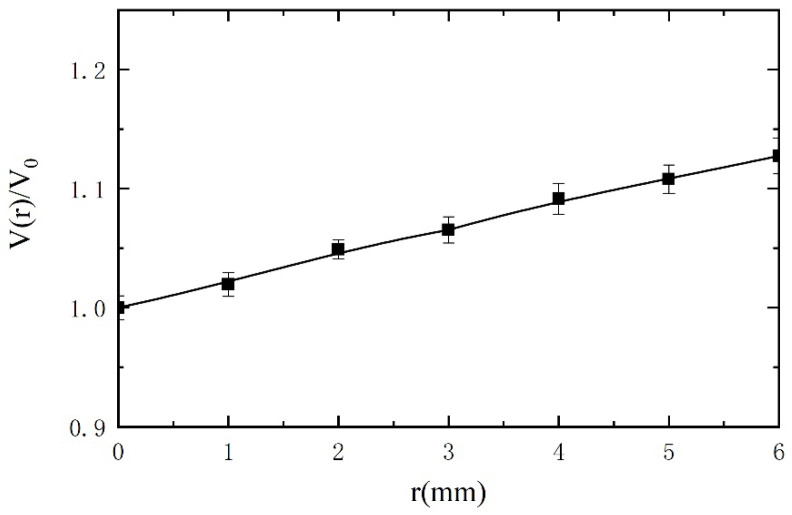
The output voltage relative to r = 0 value when metal debris passes through different radial positions.

Since the magnetic field inside the tube excited by the excitation coils is non-uniform in the radial direction, the output voltages will be different when the passing through metal debris present at different radial positions, which will lead to inaccurate estimation of the metal debris. The magnetic field distribution of the sensor is simulated by COMSOL software, and the result is shown in Figure 10. In Figure 10, the two sets of excitation coils are wound in opposite directions. The plane perpendicular to the axis of the coil is taken as the Z = 0 plane at the midpoint of a set of excitation coils. We can easily verify the non-uniform distribution of the magnetic field in the radial direction. B_0_ is the magnetic flux density at z = 0 and r = 0 (with the center of the specific excitation coils as origin). B(r) represents the magnetic flux density along the r direction in the plane of z = 0. In Figure 11, the relationship between relative magnetic flux density B(r)/B_0_ and the location on r direction is given. It can be inferred that the maximum measurement error of the sensor is about 10%. For experimental verification, a 300 μm ferrous metal debris is selected, with the same velocity but at different radial positions. The test results are shown in Figure 12. V_0_ is the voltage output when metal debris passes through the center of the sensor. It can be seen that the error caused by the difference in the radial position is within 12%. This is due to the existence of error in the experimental process, resulting in a certain difference between the experimental results and simulation results.

### 5.4. Influence of the Axial Distribution of Metal Debris on the Output Voltage

**Figure 13:**
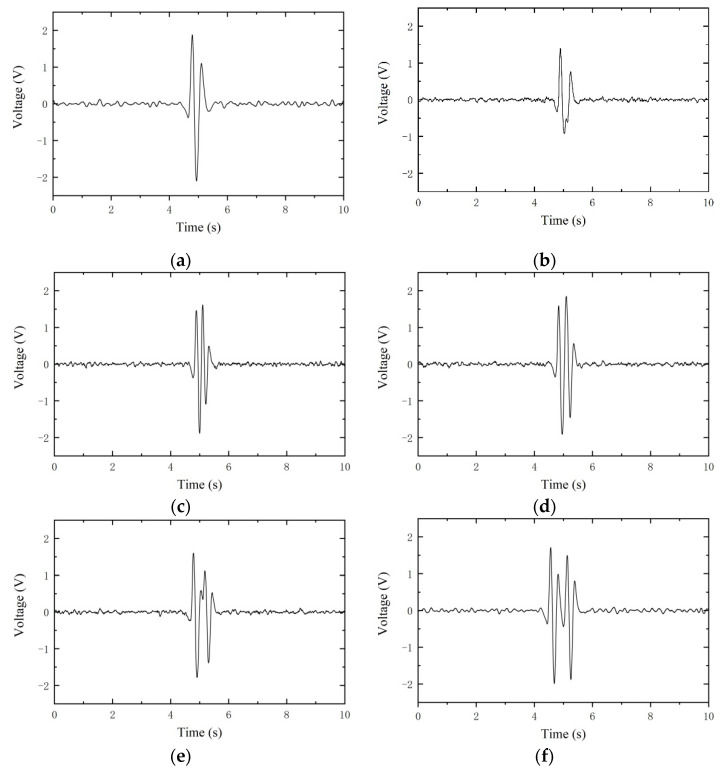
Induced voltage for two metal debris at different distances: (**a**) 15 mm; (**b**) 25 mm; (**c**) 40 mm; (**d**) 55 mm; (**e**) 70 mm; (**f**) 90 mm.

During the operation of machinery and equipment, more than one metal debris particle is produced. When the spacing between two metal debris particles is too short, the voltages they generate will be superimposed, making it difficult to recognize the true size of the metal debris. Two metal debris particles of the same size were selected for the experiment and passed through the sensor with different spacing and the same speed (0.2 m/s), and the output results are shown in Figure 13. The induced voltages of adjacent debris at different intervals are shown in Figure 13. From the experimental results, it is obvious that when the spacing is less than 25 mm, the output voltage signals are completely superimposed together, and when the spacing is greater than 90 mm, the output voltage signals are completely separated.

### 5.5. Sensor’s Speed Characteristic

**Figure 14:**
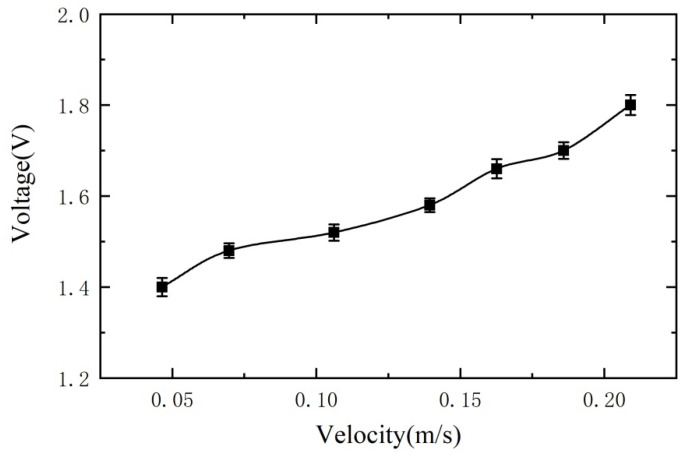
Variation of output voltage with the speed of metal debris passing through sensor.

To verify the effect of the speed of metal debris passage on the sensitivity of the sensor. We select 200 μm ferrous metal debris for the experiment. Similarly, the excitation signal is ±10 V and 125 kHz. The metal debris passes through the sensor at different speeds, and the experimental output is shown in Figure 14 (the short line indicates the standard deviation). We can see from the experimental results that the faster the metal debris passes through the sensor, the greater the voltage amplitude of the sensor output and the higher the sensitivity of the sensor.

### 5.6. Nonferrous Debris Detection Sensitivity

**Figure 15:**
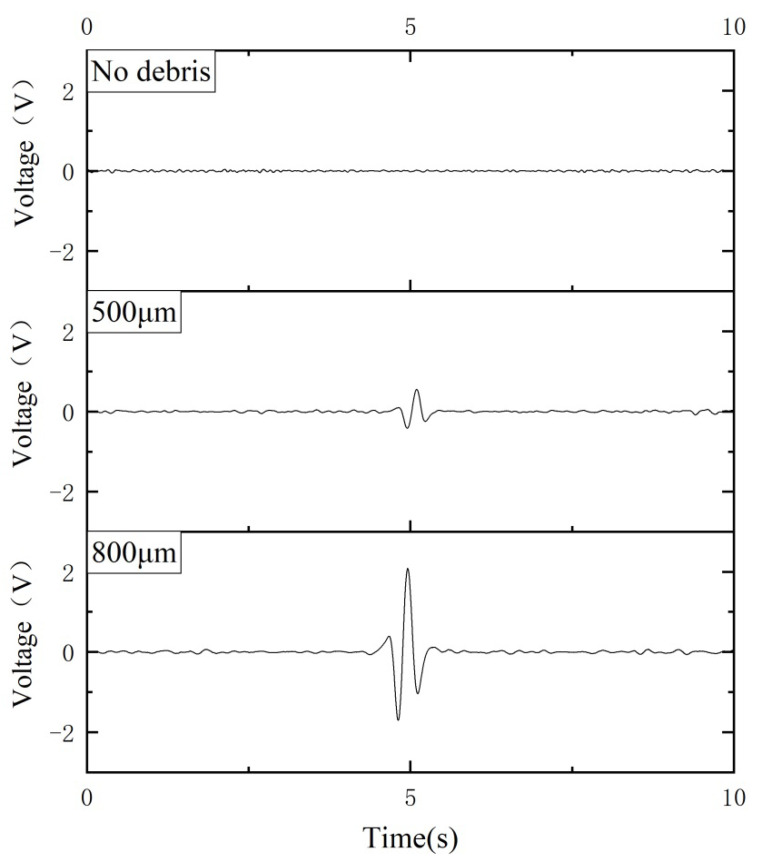
Voltage signals are generated by the passage of nonferrous metal debris of different diameters.

The ability of the sensor to detect nonferrous magnetic metal debris was also verified. Copper debris with diameters of 500 µm and 800 µm were selected for the experiments. Similarly, the excitation signal is ±10 V and 125 kHz. The velocity of the copper debris passing through the sensor is fixed as 0.2 m/s. The final output signals of the corresponding copper debris are shown in Figure 15. The experimental results clearly indicate the output signal is in opposite phase to the ferrous particle signal. Therefore, the type of particle can be identified by observing the signal phase. Assuming that the output signal amplitude is proportional to the volume of the debris, it can be deduced that the detection limit of the sensor for nonferrous is 500 μm ÷ $\sqrt[{3}]{\frac{1360}{400}}$ ≈ 313 μm (The noise level of the circuit is 400 mV, and the output voltage is 1360 mV when a copper debris with a diameter of 500 μm passes through the sensor).

## 6. Conclusions

In this paper, a novel sensor structure with dual-excitation and dual-sensing coils has been proposed for online debris monitoring. With the successful fabrication of such principle prototype, ferrous metal debris with a diameter of 115 μm and nonferrous metal debris with a diameter of 313 μm can be detected using the sensor probe with the diameter of 12.7 mm. To improve the sensor’s metal debris detection capability, the effects of the excitation frequency and radial distribution of the magnetic field on sensor sensitivity have been investigated. Results show that the highest sensitivity of the sensor has been achieved with an excitation frequency in the range of 120 to 130 kHz. Also, the radial non-uniform distribution of the magnetic field has remarkably influenced the detection accuracy by up to 12%. Furthermore, distance distribution of metal debris along the axial direction on the voltage output has been discussed. It is worth noting that the output voltage signal is completely separable when the distance between two particles is greater than 70 mm. In summary, the proposed sensor design has the ability to produce a more stable waveform and the superior performance of such device has been demonstrated throughout the experimental tests in terms of the high sensitivity. This novel sensor design also provides a useful insight into the development of high-quality sensors with superior performances. In future research, design optimization of the sensor will be conducted to improve the detection stability and precision.

## Data Availability

Data is contained within the article.
